# Conjunctival lymphangiectasia and retinal angiopathy in hereditary transthyretin amyloidosis

**DOI:** 10.1186/s40942-021-00357-x

**Published:** 2022-01-06

**Authors:** Nikhil S. Patil, Munir M. Iqbal, Lulu L. C. D. Bursztyn

**Affiliations:** 1 Michael G. DeGroote School of Medicine, McMaster University, Hamilton, ON Canada; 2 Department of Ophthalmology, Western University, London, ON Canada; 3 Clinical Neurological Sciences, Western University, London, ON Canada

**Keywords:** Asp18Glu, Transthyretin familial amyloid polyneuropathy, ATTRD18E amyloidosis, angiopathy, Ocular abnormalities

## Abstract

**Background:**

Hereditary transthyretin amyloidosis (ATTR amyloidosis) is a rare condition where a mutation in the transthyretin gene leads to systemic deposition of amyloid. The manifestations and prognosis of ATTR amyloidosis depends on the specific ATTR mutation, with over 100 mutations reported in the literature. The manifestations of many rare forms of ATTR amyloidosis have not been well described, particularly the late-onset ophthalmic findings.

**Case presentation:**

We present the case of a 43-year-old Caucasian male with a diagnosis of ATTRD18E amyloidosis confirmed by fat pad biopsy. He had diffuse systemic involvement, including cardiovascular, pulmonary, and gastrointestinal symptoms. He also had significant ocular involvement including vitreous opacities, retinal angiopathy, and conjunctival lymphangiectasia. These ocular findings modestly progressed at 2-year follow-up.

**Discussion:**

The ATTRD18E mutation is a rare variant, with few described cases. To our knowledge, this is the first documented case of ATTRD18E amyloidosis with significant ocular involvement. These ocular findings may serve as a relevant biomarker for severe disease prognosis in ATTRD18E amyloidosis. With improving treatments addressing the systemic symptoms of ATTR amyloidosis, a better understanding of the late-onset ocular symptoms is becoming increasingly relevant.

## Background

Hereditary transthyretin amyloidosis (ATTR amyloidosis) is a rare condition where a mutation in the transthyretin (ATTR) gene leads to systemic deposition of amyloid fibrils. The most common causative mutation is ATTRV30M, but over 100 mutations have been described, with the characteristics of many mutations being poorly documented due to their low prevalence [1]. ATTR amyloidosis has a heterogeneous phenotype associated with variable involvement of the heart, peripheral and autonomic nerves, kidneys, and ocular tissues [1]. Documented ocular abnormalities include vitreous opacities, dry eye, open angle glaucoma, abnormal conjunctival and retinal vessels, as well as deposition of amyloid on the iris and lens [1, 2]. The prevalence of ocular involvement increases with the duration of ATTR amyloidosis and there may be an association between the presence of vitreous amyloidosis and retinal angiopathy [2].

The manifestations and prognosis of ATTR amyloidosis appear to depend on the specific ATTR mutation. The age of onset, symptoms, as well as prevalence and penetrance of the various mutations causing ATTR amyloidosis vary with the patients’ demographic characteristics [1, 3]. There is limited literature surrounding the incidence of ATTR amyloidosis in the North American population, particularly Canada. The incidence is presumed to be very low, as evidenced by one study screening for ATTR, which found no cases from 110 patients with idiopathic neuropathy [3, 4].

We report a case of a Caucasian, Canadian male patient with a complex history of ATTR amyloidosis due to an uncommon ATTRD18E mutation, with conjunctival abnormalities and progressive retinal angiopathy.

## Case presentation

A 43-year-old Caucasian male was referred with worsening floaters in both eyes and excessive tearing in the right eye. A diagnosis of heterozygous ATTRD18E amyloidosis mutation in exon 2 had been made nearly 2 decades earlier. The patient had already experienced multiple complications of his condition, including stroke, heart failure, left ventricular thrombus, interstitial lung disease and spontaneous spinal hematoma. He was on treatment with patisiran, acetylsalicylic acid and warfarin, after prior treatment with revusiran and inotersen. His father had died at age 49 from cardiac arrest secondary to amyloidosis. The patient’s floaters and photopsias had been present for 10 years, but he had never had a formal ophthalmologic evaluation.

**Fig. 1:**
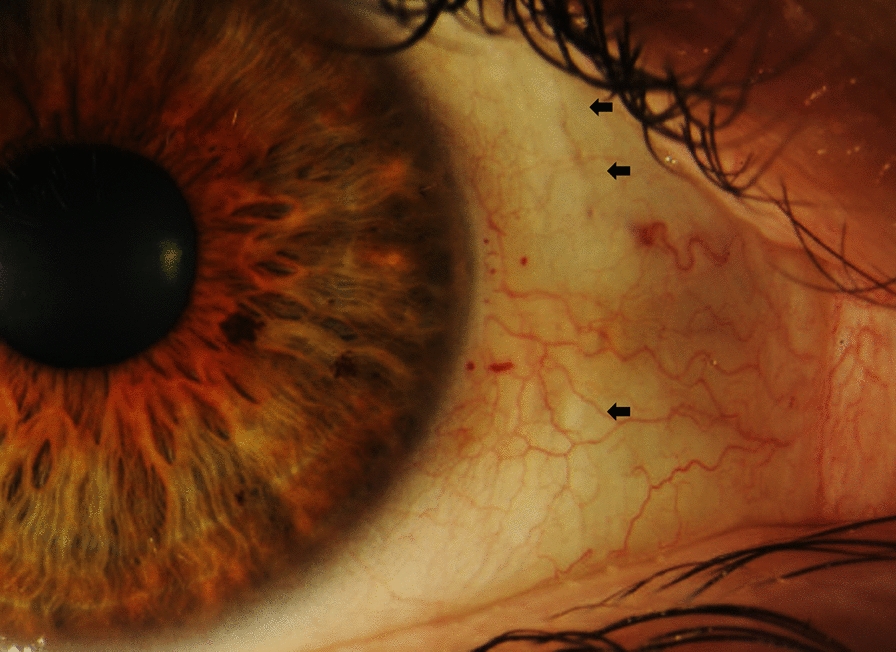
Multiple nodular, linear elevations are seen throughout the conjunctiva in both eyes, two of which are highlighted (arrows). Focal telangiectasia and dot hemorrhages are also seen at the limbus

**Fig. 2:**
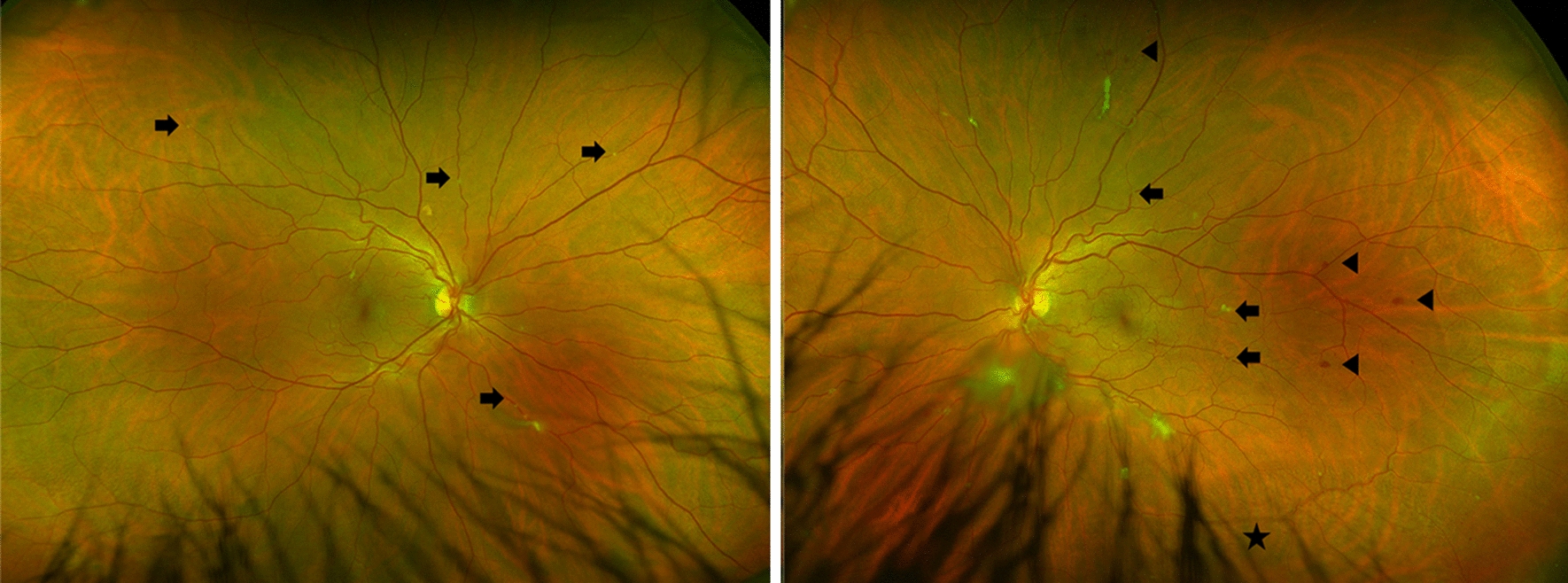
A dense vitreous opacity is noted in the left eye. Focal arterial wall amyloid deposits are noted in both eyes (arrows). In the left eye, a broad region of amyloid deposit is seen along a venous wall (star) with accompanying peripheral retinal microaneurysms (wedge)

**Fig. 3:**
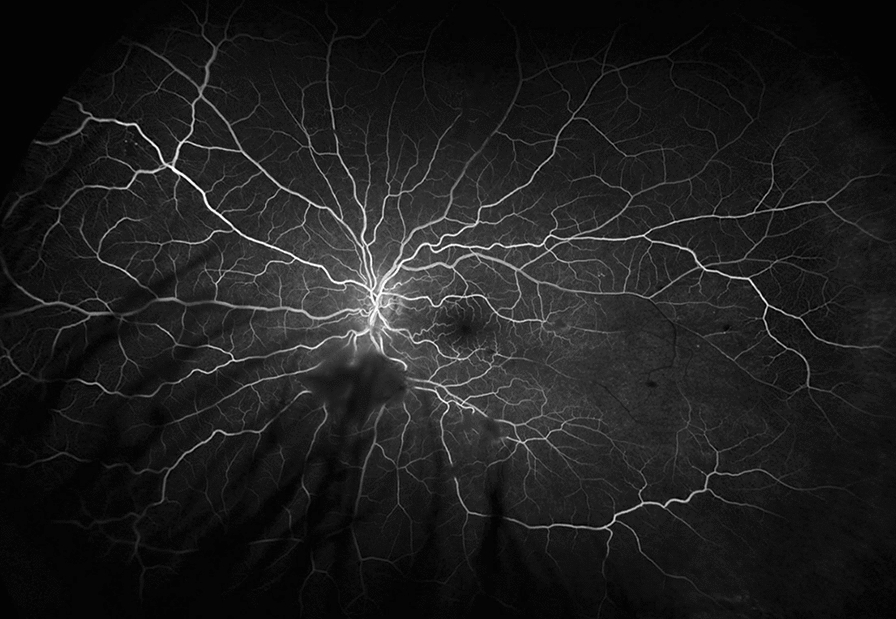
Fluorescein angiogram of the left eye shows delayed temporal arterial filling distal to the focal amyloid deposits

**Fig. 4:**
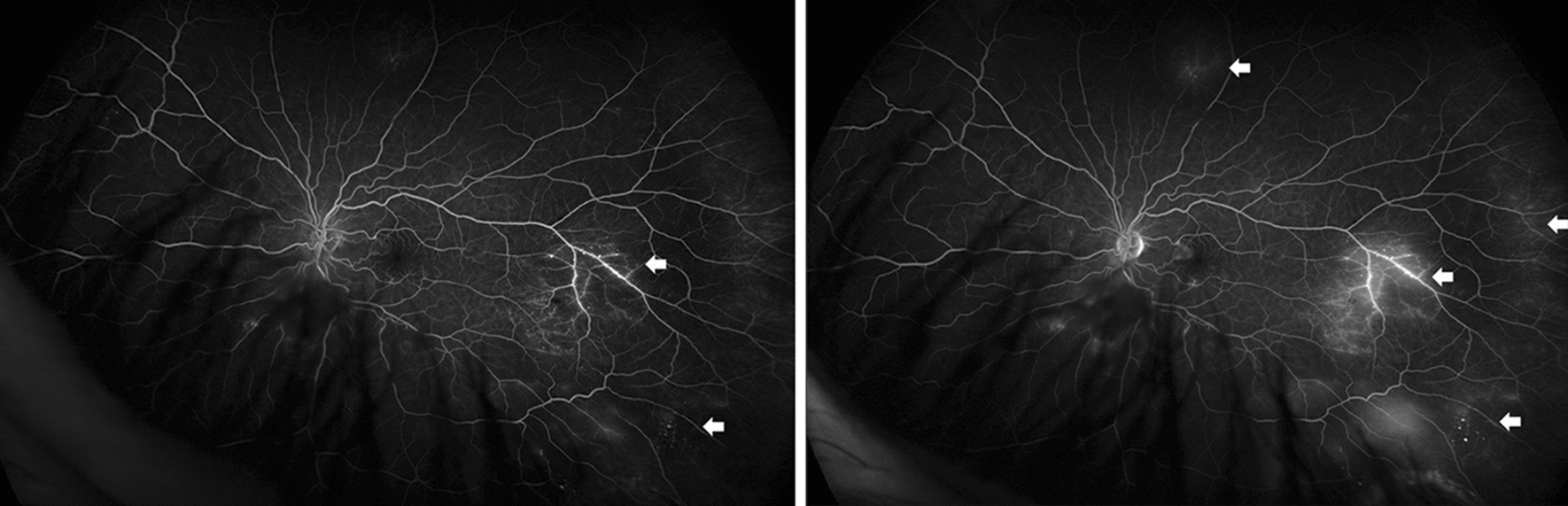
Fluorescein angiogram of the left eye shows venous telangiectatic changes in the midperiphery and periphery (arrows), followed by large and small vessel leakage late in the angiogram

**Fig. 5:**
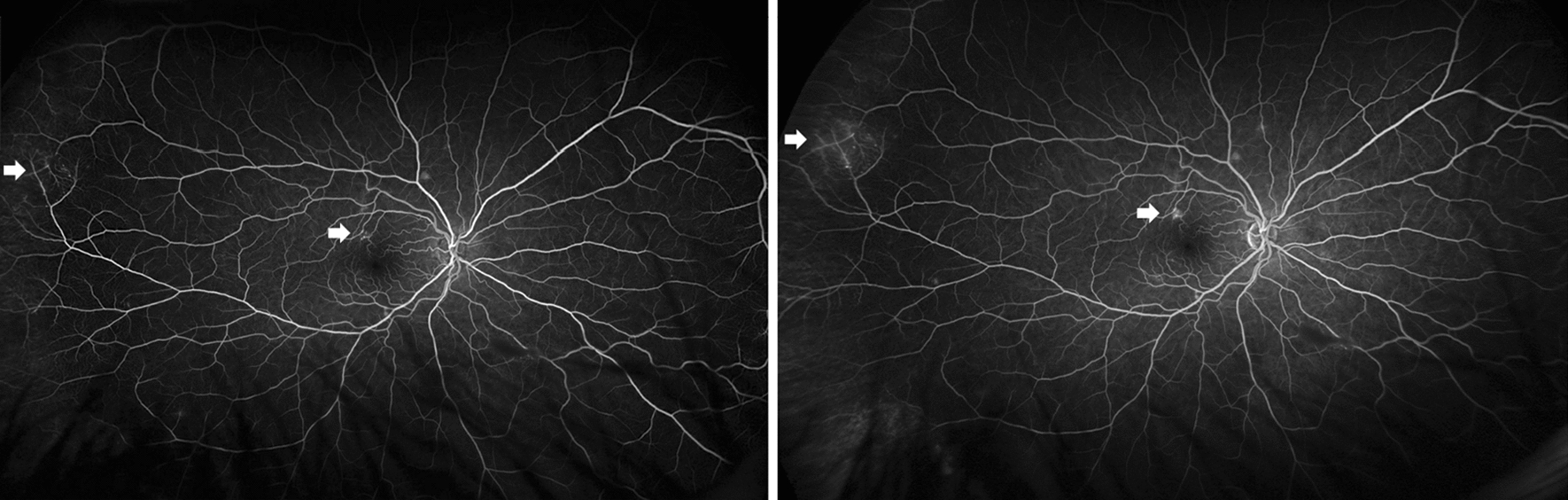
Fluorescein angiogram of the right eye shows venous telangiectatic changes in the superior macula and periphery (arrows), followed by small vessel leakage late in the angiogram

On examination, best corrected visual acuity (BCVA) of both eyes was 20/20 with normal pupils, normal intraocular pressure and no relative afferent pupillary defect. Automated visual field testing was normal in the right eye and showed a nasal defect crossing the vertical midline in the left eye. Both conjunctivae had a nodular, gelatinous appearance, consistent with conjunctival lymphangiectasia (Fig. 1). There was no evidence of amyloid deposition on the iris or lens. Dilated fundus examination revealed normal optic discs, a large sheet-like vitreous opacity in the left eye inferior to the optic disc, multiple small retinal microaneurysms, mild arterial tortuosity and focal deposits along the arterial walls, presumed to be amyloid (Fig. 2). Intravenous fluorescein angiogram (IVFA) showed delayed temporal arterial filling, inferotemporal telangiectasia and phlebitis in the left eye (Fig. 3), with late vascular leakage in the temporal retina in both eyes (Figs. 4 and 5). The location of the most prominent retinal phlebitis was felt to correspond to the visual field defect in the left eye.

**Fig. 6:**
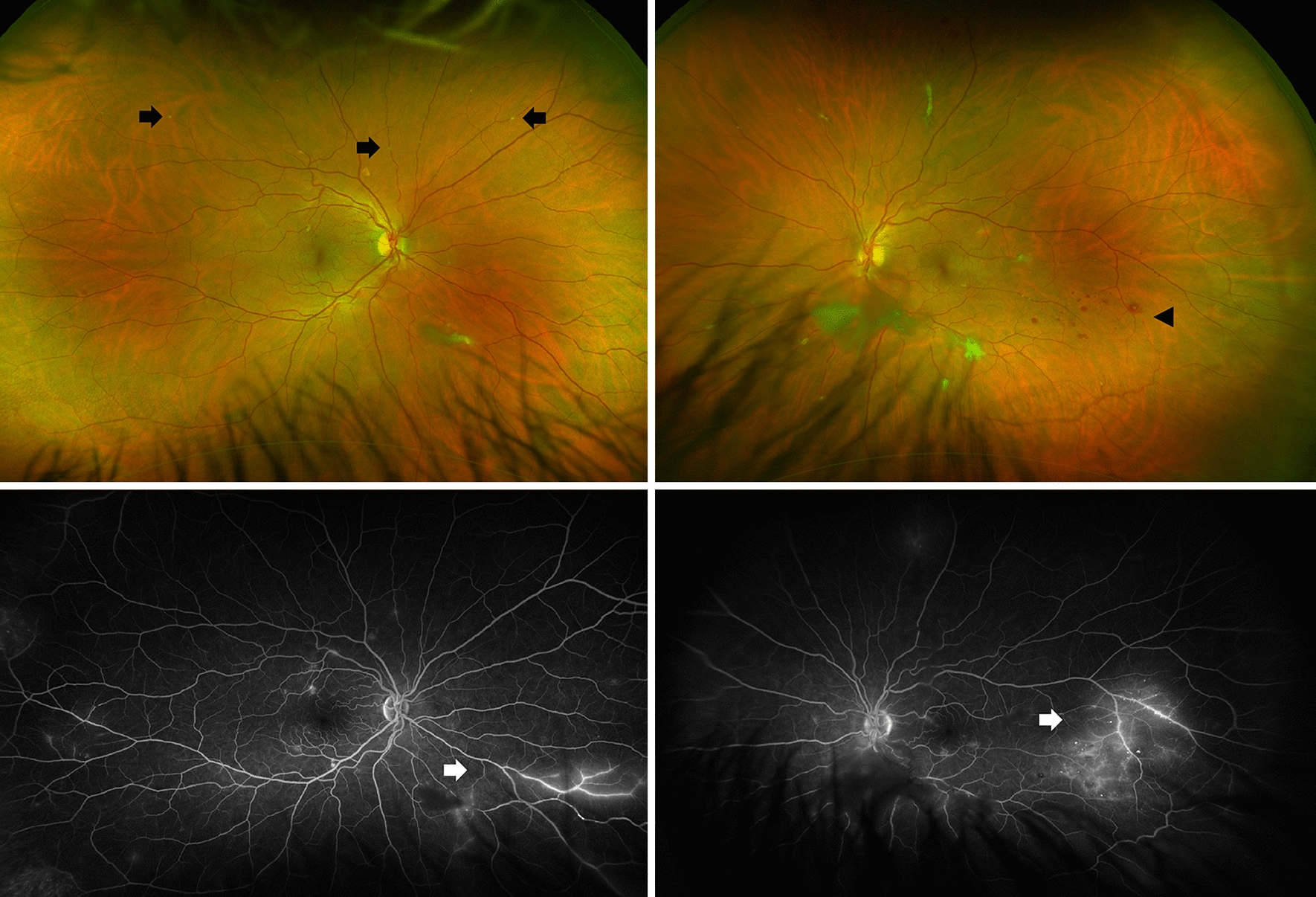
At 2-year follow up, there is increased burden of focal arterial wall amyloid deposits in both eyes (black arrows) and increased hemorrhages temporal to the macula in the left eye (wedge). Fluorescein angiogram demonstrated stable leakage temporally in the left eye and new staining nasally in the right eye (white arrows)

At follow up after 2 years, BCVA remained 20/20 in both eyes with stable visual fields, despite subjective worsening of vision. There was no change in the appearance of the optic discs. The previously observed vitreous opacities and conjunctival lymphangiectasia remained unchanged. Dilated fundus examination showed sheathing of the inferior aspect of the left optic nerve as well as areas of nonperfusion in the temporal and superior aspects of the macula. There were more hemorrhages seen temporally in the left eye. Repeat IVFA showed increased staining of the vessels as well as late leakage in both eyes, which had not previously been observed in the right eye (Fig. 6).

## Discussion and conclusions

We present a case of a Caucasian male with severe systemic disease and multiple ocular abnormalities secondary to ATTRD18E amyloidosis, including progressive retinal angiopathy. ATTRD18E amyloidosis has been rarely reported, comprising only 1/1411 patients in a large study from continental Western Europe [5]. There are few case reports describing the phenotype of this mutation [3, 6], and only one demonstrating ocular involvement, consisting of vitreous amyloid deposits [6]. To our knowledge, our patient is the first reported with significant conjunctival and retinal involvement as a result of ATTRD18E amyloidosis.

While retinal amyloid angiopathy secondary to ATTR amyloidosis is not well documented, vitreous deposition is a relatively common finding, seen in 17.4% of patients in one study [2]. Ocular pathology, such as vitreous amyloid deposits, only rarely constitutes the presenting symptoms of ATTR amyloidosis [1, 6]. In addition to the liver, ATTR is produced by the choroid plexus, retinal pigment epithelium, iris epithelium, and ciliary body [2, 7]. Mutant ATTR synthesized locally in the eye has been hypothesized to be responsible for ATTR amyloidosis related ocular pathology [2, 7].

Pharmaceutical options, such as tafamidis, patisiran, and inotersen have become increasingly available for treatment of ATTR amyloidosis. Liver transplantation was once the treatment of choice to improve longevity, but is now reserved for cases where medical treatment is unavailable or not sufficiently effective [1, 2, 4]. The persistence and progression of pupillary border irregularities, vitreous opacities, and glaucoma following either liver transplantation or medical treatment suggest that they are the result of independent synthesis of mutant ATTR by the RPE and ciliary body rather than the liver [1, 2, 4, 7]. It is not yet clear if amyloid deposition in retinal vessels is the result of local production by the RPE or circulating mutant ATTR [8]. With the increased longevity experienced as a result of liver transplantation and new medical treatments, the incidence of ocular pathologies may rise and more frequently require management [2].

Reported cases of retinal involvement secondary to ATTR amyloidosis describe retinal telangiectasias, retinal microangiopathy, and tortuous retinal vessels [2, 9–11]. Retinal ischemia may lead to neovascularization with secondary complications such as glaucoma or vitreous hemorrhage. The retinal angiopathy in our case is relatively extensive compared to most documented cases of retinal involvement in ATTR amyloidosis, and was associated with a severe disease course and conjunctival lymphangiectasia.

Conjunctival lymphangiectasia associated with ATTR was first described in 2009 in three patients with ATTRS77Y amyloidosis, a relatively common variant [7]. A subsequent cross-sectional study revealed conjunctival lymphangiectasia in 13/24 patients (54%) with ATTRS77Y amyloidosis. This finding correlated with more severe neurologic and cardiac dysfunction and was felt to be a biomarker for severe systemic disease in this genotype [7]. As our patient also had severe systemic disease, the presence of conjunctival lymphangiectasia as a biomarker may also be relevant in ATTRD18E amyloidosis or other mutations.

In summary, we describe a patient with ATTRD18E amyloidosis, demonstrating conjunctival lymphangiectasia and retinal angiopathy, neither of which have previously been reported in this disease variant. As treatment for ATTR amyloidosis advances, patients are living longer and the importance of detecting, monitoring, and treating ocular manifestations becomes more crucial, particularly as current medical and surgical treatment options do not address ocular symptoms [1, 2, 4]. Further investigation and reporting of the retinal manifestations of ATTR amyloidosis is warranted to better understand the pathogenesis, incidence, and prognosis of retinal abnormalities.

## Data Availability

Not applicable.
